# Psychiatric Disorders and Cardiovascular Diseases During the Diagnostic Workup of Suspected Prostate Cancer

**DOI:** 10.1093/jncics/pkaa108

**Published:** 2020-11-07

**Authors:** Qing Shen, Yuanjun Ma, Anna Jöud, Maria E C Schelin, Katja Fall, Ove Andrén, Fang Fang

**Affiliations:** 1 Unit of Integrative Epidemiology, Institute of Environmental Medicine, Karolinska Institutet, Stockholm, Sweden; 2 Department of Oncology-Pathology, Karolinska Institutet, Stockholm, Sweden; 3 Division of Occupational and Environmental Medicine, Department of Laboratory Medicine Lund, Lund University, Lund, Sweden; 4 Clinical Epidemiology and Biostatistics, School of Medical Sciences, Örebro University, Örebro, Sweden; 5 Department of Urology, School of Medical Sciences, Örebro University, Örebro, Sweden

## Abstract

**Background:**

It is unknown whether the rate of psychiatric disorders and cardiovascular disease increases during the diagnostic workup of suspected prostate cancer.

**Methods:**

We designed a population-based cohort study including 579 992 men living during 2005-2014 in Skåne, Sweden, according to the Swedish Total Population Register and the Skåne Healthcare Register (SHR). We used the Swedish Cancer Register and the SHR to identify all men with a new diagnosis of prostate cancer (N = 10 996), and all men underwent a prostate biopsy without receiving a cancer diagnosis (biopsy group, N = 20 482) as exposed to a diagnostic workup. Using Poisson regression, we compared the rates of psychiatric disorders and cardiovascular disease during the period before diagnosis or biopsy of exposed men with the corresponding rates of unexposed men.

**Results:**

We found an increased rate of psychiatric disorders during the period before diagnosis or biopsy among men with prostate cancer (incidence rate ratio [IRR] = 1.87, 95% confidence interval [CI] = 1.67 to 2.10) and men in the biopsy group (IRR = 2.22, 95% CI = 2.08 to 2.37). The rate of cardiovascular disease increased during the period before diagnosis or biopsy among men with prostate cancer (IRR = 2.22, 95% CI = 2.12 to 2.32) and men in the biopsy group (IRR = 2.56, 95% CI = 2.49 to 2.63). Greater rate increases were noted for a diagnostic workup due to symptoms than due to other reasons.

**Conclusions:**

There was an increased risk of psychiatric disorders and cardiovascular disease during the diagnostic workup of suspected prostate cancer regardless of the final cancer diagnosis.

Prostate cancer is the most common cancer and the second leading cause of cancer death among men in Europe and the United States (1). Receiving a diagnosis of prostate cancer is stressful and may lead to adverse health outcomes, including suicide (2). Active treatment for prostate cancer might introduce side effects, including urination and bowel problems, pain, and sexual dysfunction (3,4), whereas in case of active surveillance or “watchful waiting,” patients have to live with the uncertainty of cancer progression and spread, contributing further to psychological distress and anxiety (5,6). Recent studies have, for example, shown that men with prostate cancer have increased risk of psychiatric disorders, suicidal behaviors, and cardiovascular disease (7-11).

The severe psychological distress is, however, not only present after receiving a final diagnosis of prostate cancer. Patients reported an elevated level of psychological distress already during the diagnostic workup of prostate cancer, especially while waiting for the biopsy result (12,13). Because of the increasingly common practice of prostate diagnostic workup due to either symptoms or screening such as the prostate-specific antigen (PSA) test findings, the potential health impact of prostate diagnostic workup applies not only to men who are finally diagnosed with prostate cancer but also to a larger population of men who are evaluated for, but never diagnosed with, prostate cancer. Therefore, we performed this population-based study to evaluate the risks of psychiatric disorders and cardiovascular disease among men who underwent a diagnostic workup for suspected prostate cancer between 2005 and 2014 in Skåne, Sweden.

## Methods

### Study Design

Skåne is the south region of Sweden with about 1.3 million residents. The Skåne Healthcare Register (SHR) contains information on all levels of health care provided in Skåne, including dates of health-care visit and diagnostic and procedure codes (surgical and nonsurgical), from 2004 onward (14). It has virtually complete coverage of both inpatient and outpatient specialist care and primary care. The Swedish Cancer Register was initiated in 1958 with a national completeness approaching 100% (15,16). Through cross-linking the Swedish Total Population Register to these registers using the individually unique personal identification numbers, we conducted a cohort study following 579 992 men living in Skåne and with at least 1 health-care visit registered in the SHR from January 1, 2005, or 18th birthday, whichever came later, until a diagnosis of prostate cancer, a diagnosis of any other cancer, death, emigration out of Skåne, or December 31, 2014, whichever came first.

Within the study cohort, we first identified all men who had a prostate cancer diagnosis (according to the Swedish Cancer Register) during the follow-up (ie, prostate cancer patients). Among the remaining men, we identified those who had undergone a prostate biopsy without a diagnosis of prostate cancer (ie, biopsy group, according to the Swedish Cancer Register and SHR). All these men were defined as exposed to a diagnostic workup for suspected prostate cancer. We identified in total 10 996 men with a newly diagnosed prostate cancer and 20 482 men who underwent a prostate biopsy without receiving a cancer diagnosis (biopsy group). For men with multiple prostate biopsies during the follow-up (35.7% of men in the biopsy group had up to 4 biopsies), we studied only the first biopsy for each man.

The median waiting time from the first specialist referral to primary treatment initiation was approximately 180 days for a majority of the prostate cancer patients in Skåne (17). In our data, prostate cancer patients started to have a statistically significantly increased number of health-care visits from the 11th week before diagnosis (Supplementary Figure 1, available online). We therefore assigned the first health-care visit within 3 months before the date of diagnosis (for prostate cancer patients) or biopsy (for biopsy group) as the start of the diagnostic workup. If there was no recorded health-care visit during the 3 months, we assigned the 90th day before diagnosis or biopsy as the start of workup (11.6% of prostate cancer patients and 12.3% of men in biopsy group). We estimated the rates of psychiatric disorders and cardiovascular disease during the time period before diagnosis (ie, the interval between the start of workup and the day before diagnosis or biopsy) among men who had a prostate diagnostic workup. The date of biopsy overlapped with the date of diagnosis for the majority of the patients with prostate cancer.

We estimated the rates of psychiatric disorders and cardiovascular disease during a reference period. Men without a diagnostic workup for suspected prostate cancer during the follow-up contributed all their person-time to the reference period. Men with a diagnostic workup for suspected prostate cancer contributed also their person-time to the reference period before the start of workup.

The study was approved by the Regional Ethical Review Board in Stockholm, Sweden.

### Ascertainment of Psychiatric Disorders and Cardiovascular Disease

We used the 10th Swedish revision of the International Classification of Diseases (ICD) codes F10-F99 to identify all diagnoses of psychiatric disorders and I00-I99 to identify all diagnoses of cardiovascular disease through either primary or specialist care, according to the SHR. Consecutive events that occurred within 28 days of each other were considered as 1 event. Deaths due to cardiovascular disease identified from the Causes of Death Register that were not preceded by a related hospital visit were also considered as an outcome of interest. We classified psychiatric disorders as stress reaction or adjustment disorder, depression, anxiety, substance abuse, and other psychiatric disorders (18). We classified cardiovascular disease as myocardial infarction, other diseases of the heart, embolism or thrombosis, stroke, and other diseases of the circulatory system (7) (Supplementary Table 1, available online).

### Statistical Analysis

We first described the characteristics of men with and without a prostate diagnostic workup, including age at the start of workup, reason for workup, surgical treatment, cohabitating status, preexisting psychiatric disorders and cardiovascular disease, and tumor stage (for cancer patients alone).

We calculated the crude incidence rates (IRs) of psychiatric disorders and cardiovascular disease among the exposed men by dividing the number of events by accumulated person-months at risk. We used Poisson regression to calculate incidence rate ratios (IRRs) and their 95% confidence intervals (CIs) by comparing the IRs of exposed men with the IRs of the reference group. We used attained age as the underlying timescale and split the timescale to allow variances by month and a clustered sandwich estimator to account for the intra-individual correlation. All the analyses were additionally adjusted for time since start of follow-up, cohabitating status, registered parish as a proxy for socioeconomic status (obtained from the Swedish Total Population Register), and preexisting psychiatric disorders or cardiovascular disease. Information on preexisting diseases was ascertained from the SHR from 2004 onward using the 10th Swedish revision of the ICD codes F10-F99 for psychiatric disorders and I00-I99 for cardiovascular disease as described above, and updated for each month to account for the time-varying nature of these covariables.

We separately analyzed diagnostic workup initiated by symptoms and diagnostic workups due to other reasons. A diagnostic workup starting from a prostate-related health-care visit was considered as a workup due to symptoms. ICD codes used to identify a possible prostate-related health-care visit were listed in Supplementary Table 2 (available online). We also conducted subgroup analyses by age, calendar period, cohabitating status, and preexisting psychiatric disorders or cardiovascular disease. Because frequency of health-care visits might be associated both with the possibility of undergoing a diagnostic workup and the risk of being diagnosed with psychiatric disorders or cardiovascular disease, we further adjusted the analyses for the frequency of health-care visit per time period. The frequency was calculated as the number of health-care visits divided by total number of days per time period. Finally, to assess the potential influence of residual confounding, we performed a within-individual comparison using conditional Poisson regression (19), comparing the rates of psychiatric disorders and cardiovascular disease during the time period between start of workup and the day before diagnosis or biopsy with the rates during the follow-up before the start of workup among the exposed men. All person-time accumulated during the follow-up time before the start of workup was contributed to the reference period, and the mean duration was more than 4 years. To study the risks of psychiatric disorders and cardiovascular disease in relation to multiple prostate biopsies, we conducted a within-individual comparison among men with repeated biopsies during follow-up using the same definitions for diagnostic workup and reference period as in main analysis.

The assumption of equal-dispersion for Poisson regression was found to hold for all analyses. All analyses were conducted in SAS 9.4 (SAS Institute) and Stata 16.1 (StataCorp LP). The level of statistical significance was defined as a 2-sided *P* less than .05 in Supplementary Figure 1 (available online) using the χ^2^ test.

## Results

### Study Population Characteristics

The mean age at the start of diagnostic workup was 65.3 years for men in the biopsy group and 68.3 years for prostate cancer patients (Table 1). A total of 45.1% of the men in the biopsy group and 45.3% of the cancer patients underwent a diagnostic workup due to symptoms. Patients with prostate cancer were more likely to be cohabitating and less likely to have preexisting psychiatric disorders or cardiovascular disease than men of the biopsy group.

**Table 1.** Characteristics of men without a prostate diagnostic workup and men with a prostate diagnostic workup by their final diagnosis in a population-based cohort study during 2005-2014 in Skåne, Sweden^a^

Characteristics	Men without prostate diagnostic workup	Biopsy group	Prostate cancer patients
Men, No.	548 514	20 482	10 996
Age at follow-up/workup start, mean (SD), y	40.0 (18.5)	65.3 (12.4)	68.3 (9.0)
Tumor stage, No. (%)			
T0M0N0 + stage I + II	—	—	8178 (74.4)
Stage III	—	—	1197 (10.9)
Stage IV	—	—	990 (9.0)
Missing TMN	—	—	631 (5.7)
Reason for diagnostic workup, No. (%)			
Symptoms	—	9236 (45.1)	4982 (45.3)
Others	—	11 246 (54.9)	6014 (54.7)
Surgical treatment, No. (%)			
Yes	—	1288 (6.3)	3338 (30.4)
No	—	19 194 (93.7)	7658 (69.6)
Cohabitation status, No. (%)			
Cohabitating	202 030 (26.8)	12 956 (63.3)	7419 (67.5)
Noncohabitating	346 484 (63.2)	7526 (36.7)	3577 (32.5)
Preexisting psychiatric disorder, No. (%)			
Yes	32 251 (5.9)	3822 (18.7)	1528 (13.9)
No	516 263 (94.1)	16 660 (81.3)	9468 (86.1)
Preexisting cardiovascular disease, No. (%)			
Yes	38 607 (7.0)	10 953 (53.5)	5432 (49.4)
No	509 907 (93)	9529 (46.5)	5564 (50.6)

a TMN = Tumor, Node, Metastasis.

### Psychiatric Disorders

**Figure 1.**
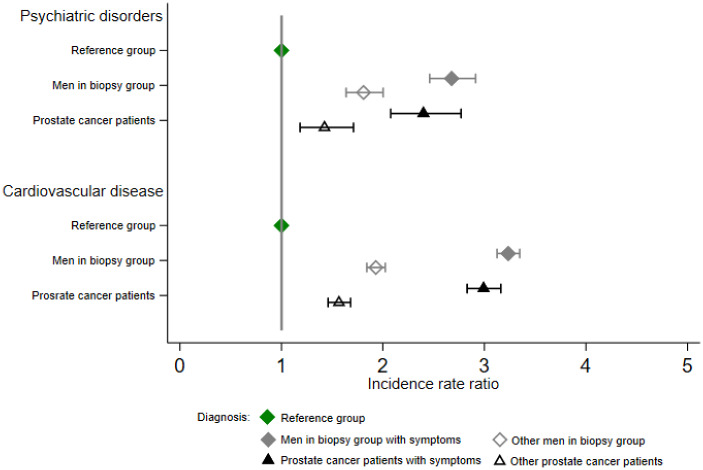
Incidence rate ratios and 95% confidence intervals of psychiatric disorders and cardiovascular disease during the period before diagnosis of men who underwent a prostate diagnostic workup by whether the workup was due to symptoms in a population-based cohort study during 2005-2014 in Skåne, Sweden.

Compared with the reference group, we found a higher rate of psychiatric disorders during the period before biopsy for the biopsy group (incidence rate (IR) = 28.18 per 1000 person-months, IRR = 2.22, 95% CI = 2.08 to 2.37) and before diagnosis for prostate cancer patients (IR = 18.86 per 1000 person-months, IRR = 1.87, 95% CI = 1.67 to 2.10) (Table 2). Among patients with prostate cancer, the rate increment was of a similar magnitude across different tumor stages. The rate increase was, however, more pronounced among patients with a diagnostic workup due to symptoms compared with patients with a diagnostic workup due to other reasons (Figure 1). A rate increment was noted for most individual psychiatric disorders and among men in the biopsy group and prostate cancer patients (Table 3). The increased rate of psychiatric disorders was more pronounced among younger patients, patients diagnosed in earlier calendar periods, and patients with preexisting psychiatric disorders (Supplementary Table 3, available online). The estimate was reduced but did not disappear after further adjustment for frequency of health-care visit (Supplementary Table 4, available online). We found similar results in the within-individual comparison (Table 4). The rate increase was also noted among men with repeated biopsies (Supplementary Table 5, available online).

**Table 2.** IRs (per 1000 person-months) and IRRs of psychiatric disorders and cardiovascular disease during the period before diagnosis of men who underwent a prostate diagnostic workup in a population-based cohort study during 2005-2014 in Skåne, Sweden

Men	Events, No.	Crude IR	IRR (95% CI)^a^
Psychiatric disorders			
Reference group^b^	607 864	11.50	1.00
Biopsy group	1051	28.18	2.22 (2.08 to 2.37)
Prostate cancer patients	343	18.86	1.87 (1.67 to 2.10)
T0M0N0 + stage I + II	249	17.61	1.78 (1.56 to 2.03)
Stage III	41	23.05	2.30 (1.65 to 3.22)
Stage IV	33	26.32	2.63 (1.86 to 3.72)
Missing stage	20	19.76	1.57 (0.91 to 2.72)
Cardiovascular diseases			
Reference group^b^	1 019 516	19.30	1.00
Biopsy group	5182	138.90	2.56 (2.49 to 2.63)
Prostate cancer patients	2053	112.97	2.22 (2.12 to 2.32)
T0M0N0 + stage I + II	1377	97.38	2.06 (1.95 to 2.17)
Stage III	281	158.08	2.20 (1.96 to 2.48)
Stage IV	285	228.98	3.50 (3.09 to 3.96)
Missing stage	110	108.87	2.25 (1.85 to 2.73)

a All the analyses were adjusted for age, cohabitating status, registered parish, and preexisting psychiatric disorders or cardiovascular disease. CI = confidence interval; IR = incidence rate; IRR = incidence rate ratio.

b Reference group included person-time accumulated from men who did not have any prostate diagnostic workup during the follow-up and the person-time accumulated before the start of workup from men with a prostate diagnostic workup during the follow-up.

**Table 3.** IRs (per 1000 person-months) and IRRs of individual psychiatric disorders and cardiovascular disease during the period before diagnosis of men who had a prostate diagnostic workup in a population-based cohort study during 2005–2014 in Skåne, Sweden Stress reaction or adjustment disorder

Outcome subtypes	Reference group^a^			Biopsy group			Prostate cancer patients		
	No.	Crude IR	IRR	No.	Crude IR	IRR (95% CI)^b^	No.	Crude IR	IRR (95% CI)^b^
Psychiatric disorders									
Stress reaction or adjustment disorder	66 723	1.26	1.00	80	2.14	1.81 (1.43 to 2.30)	18	0.99	1.06 (0.65 to 1.72)
Depression	142 913	2.70	1.00	286	7.67	2.25 (1.99 to 2.55)	100	5.50	1.98 (1.60 to 2.46)
Anxiety	103 318	1.96	1.00	163	4.37	2.39 (2.03 to 2.81)	47	2.58	1.86 (1.37 to 2.53)
Substance abuse	109 207	2.07	1.00	167	4.48	1.66 (1.41 to 1.95)	75	4.12	1.86 (1.47 to 2.36)
Other psychiatric disorders	185 703	3.51	1.00	355	9.52	2.64 (2.36 to 2.95)	103	5.66	2.05 (1.67 to 2.53)
Cardiovascular disease									
Myocardial infarction	25 944	0.49	1.00	94	2.52	1.81 (1.45 to 2.26)	15	0.83	0.61 (0.37 to 1.01)
Other diseases of the heart	400 672	7.58	1.00	2318	62.13	3.03 (2.91 to 3.16)	1058	58.22	3.02 (2.85 to 3.21)
Embolism or thrombosis	11 567	0.22	1.00	97	2.60	4.78 (3.81 to 5.98)	34	1.87	3.67 (2.55 to 5.28)
Stroke	34 507	0.65	1.00	161	4.32	2.29 (1.92 to 2.73)	48	2.64	1.48 (1.08 to 2.02)
Other diseases of the circulatory system	546 826	10.35	1.00	2512	67.33	2.25 (2.16 to 2.35)	898	49.41	1.77 (1.64 to 1.90)

a Reference group included person-time accumulated from men who did not have any prostate diagnostic workup during the follow-up and the person-time accumulated before the start of workup from men with a prostate diagnostic workup during the follow-up. CI = confidence interval; IR = incidence rate; IRR = incidence rate ratio.

b All the analyses were adjusted for age, cohabitating status, registered parish, and preexisting psychiatric disorders or cardiovascular disease.

**Table 4.** IRs (per 1000 person-months) and IRRs of psychiatric disorders and cardiovascular disease during the period before diagnosis of men who had a prostate diagnostic workup during 2005–2014 in Skåne, Sweden (within-individual comparison)

Men	Events, No.	Crude IR	IRR (95% CI)^a^
Psychiatric disorders			
Reference period^b^	16,325	8.81	1.00
Biopsy group	1,051	28.18	2.19 (2.02 to 2.37)
Prostate cancer patients	343	18.86	2.15 (1.88 to 2.47)
T0M0N0 + stage I + II	249	17.61	2.04 (1.75 to 2.39)
Stage III	41	23.05	2.49 (1.65 to 3.77)
Stage IV	33	26.32	3.43 (2.20 to 5.36)
Missing stage	20	19.76	1.73 (0.99 to 3.02)
Cardiovascular disease			
Reference period^b^	70 427	38.02	1.00
Biopsy group	5182	138.90	2.38 (2.30 to 2.47)
Prostate cancer patients	2053	112.97	2.46 (2.33 to 2.60)
T0M0N0 + stage I + II	1377	97.38	2.27 (2.13 to 2.42)
Stage III	281	158.08	2.55 (2.22 to 2.91)
Stage IV	285	228.98	3.91 (3.32 to 4.61)
Missing stage	110	108.87	2.35 (1.88 to 2.95)

a CI = confidence interval; IR = incidence rate; IRR = incidence rate ratio.

b Reference group included person-time accumulated from men who did not have any prostate diagnostic workup during the follow-up and the person-time accumulated before the start of workup from men with a prostate diagnostic workup during the follow-up.

### Cardiovascular Disease

Compared with the reference group, we found a higher rate of cardiovascular disease during diagnostic workup for both men in the biopsy group (IR = 138.90 per 1000 person-months, IRR = 2.56, 95% CI = 2.49 to 2.63) and prostate cancer patients (IR = 112.97 per 1000 person-months, IRR = 2.22, 95% CI = 2.12 to 2.32) (Table 2). Among patients with prostate cancer, the magnitude of rate increase was similar across different tumor stages, with a slightly greater rate increase noted among men with tumor stage IV. Similar to psychiatric disorders, the increased rate was more pronounced for men with a workup due to symptoms than for men with a workup due to other reasons (Figure 1). The increased rate of cardiovascular disease was noted for a majority of the individual cardiovascular diseases studied, especially embolism or thrombosis (Table 3). The increased rate of cardiovascular disease was more pronounced among younger patients, patients diagnosed in earlier calendar periods, and patients with preexisting cardiovascular disease (Supplementary Table 3, available online). The result diminished slightly after further adjustment for frequency of health-care visit (Supplementary Table 4, available online). We also found similar results of rate increment during the diagnostic workup for both men in the biopsy group and prostate cancer patients in the within-individual comparison (Table 4). A similar rate increase was also observed among men with repeated biopsies during follow-up (Supplementary Table 5, available online).

## Discussion

Our study is the first, to our knowledge, to demonstrate an increased risk of psychiatric disorders and cardiovascular disease during the diagnostic workup of suspected prostate cancer, with a focus on the time period before receiving a definite diagnosis, among both prostate cancer patients and men who underwent a prostate biopsy without receiving a diagnosis of prostate cancer. This finding suggests that the diagnostic workup for suspected prostate cancer may take a considerable toll on the health and well-being of those who are vulnerable. Our study provides novel and clear evidence for the need of monitoring and prevention of psychiatric and cardiovascular morbidities during clinical evaluation of suspected prostate cancer and calls for caution in implementing screening programs for prostate cancer in a wide-reaching population.

Previous studies have mostly examined the risk of psychiatric disorders and cardiovascular disease among patients with prostate cancer, primarily after treatment. For example, prostate cancer patients were shown to have increased prevalence of depression and anxiety from time of diagnosis and through the posttreatment survivorship (20). The burden of such psychiatric disorders was shown to be related to the specific type of treatment (21-23). Similarly, men with prostate cancer were shown to have an increased risk of cardiovascular disease, especially after endocrine treatment (24). Androgen deprivation therapy was also shown to be related to a higher risk of cardiovascular disease (25). In contrast, relatively little is known about the risks of psychiatric disorders and cardiovascular disease around the time of diagnosis, especially during the clinical evaluation of potential prostate cancer. Potential health consequences of diagnostic workup should not be overlooked, because a greater number of men are indeed evaluated for suspected prostate cancer than those who will eventually receive a diagnosis of prostate cancer. In our study, twice as many men received a negative prostate biopsy than men who were finally diagnosed with prostate cancer.

Two clinical studies have assessed self-reported psychological distress during the clinical investigation of suspected prostate cancer, demonstrating the highest distress level while waiting for biopsy result (12,26). A higher-than-expected prevalence of anxiety was also noted in relation to PSA testing among men not later diagnosed with cancer (27). However, available clinical studies have so far not been population based and included relatively small number of patients, whereas men participating in PSA testing are most likely healthier than average and not representative of the entire male population. Furthermore, most of the studies employed self-reported assessment of psychological distress or psychiatric symptoms, leaving concrete health outcomes related to psychological stress unraveled. Our findings of increased risks of psychiatric disorders and cardiovascular disease during the prostate diagnostic workup, especially the time before diagnosis, are an extension of these findings. We noted a similar rate increase of psychiatric disorders and cardiovascular disease among men with different tumor stages, suggesting a sustained high level of psychological distress regardless of disease severity.

Using a population-based cohort study including half a million men with a follow-up of 10 years, we had the unique opportunity to assess the risks of clinically diagnosed psychiatric disorders and cardiovascular disease during the diagnostic workup of suspected prostate cancer. The population-based design, complete follow-up, and prospectively and independently collected information on the exposure and outcomes alleviated greatly the concern of selection and information biases. Another main strength of the study is the possibility to contrast the risk during the diagnostic workup that led to a diagnosis of prostate cancer with the risk during a diagnostic workup that did not lead to a cancer diagnosis. Finally, the largely similar results observed between the main analyses with careful adjustment of a good number of potential confounders and the within-individual comparison helped to further alleviate concerns about residual confounding due to unknown or unmeasured differences between the comparison groups.

Nevertheless, potential limitations of this study should also be acknowledged. Individuals under clinical evaluation for a potential cancer have closer access to healthcare and a higher risk of being diagnosed with other diseases. Such potential surveillance bias would, however, be less applicable to acute cardiovascular events, such as myocardial infarction. It is also possible that the increased risk of cardiovascular disease during the period before biopsy or diagnosis might to some extent be attributed to clinical practice (eg, cardiac clearance) for the safety of invasive procedures (eg, biopsy). Secondly, men of the exposed group (prostate cancer patients and the biopsy group) were older and had a different status of cohabitation and history of preexisting psychiatric disorders and cardiovascular disease compared with men in the reference group. We carefully controlled for these variables in all analyses and additionally performed stratified analyses using these variables. The similar results obtained in the stratified analyses and the within-individual analyses argue against, however, that the observed associations were mainly attributable to these factors.

We had no information on PSA testing status of the study participants. As a result, we could only separate the prostate diagnostic workup as due to symptoms or due to other reasons, depending on whether there was a prostate-related health-care visit during the 3 months before biopsy or prostate cancer diagnosis. It would have been interesting to separately study prostate diagnostic workup due to abnormal findings from PSA testing. The greater magnitude in risk increases of psychiatric disorders and cardiovascular disease noted during a symptom-based workup, compared with a workup due to other reasons, suggests that the corresponding risk increases during a prostate diagnostic workup due to abnormal PSA findings are likely of smaller magnitude. This is likely because cancer patients diagnosed through screening usually have lower tumor stage and a better prognosis than symptomatic patients (28). Further, although we were able to classify men with prostate cancer by tumor stage, we had little information on other cancer characteristics (eg, Gleason score) and could not assess whether the risks of psychiatric disorders and cardiovascular disease would also differ by other cancer characteristics. Finally, although severe psychological distress experienced during the diagnostic workup of a suspected prostate cancer could be 1 important contributor to the observed risk increment, as we hypothesized, it is unlikely to be the only one. In addition to surveillance bias as discussed above, other factors could also have contributed to the observed results. For instance, cancer-related inflammation may have triggered the debut of psychiatric symptoms and contributed to the increased risk of psychiatric disorders (29,30), and cancer-related cardiometabolic alterations might have initiated the debut of cardiovascular symptoms and contributed to the increased risks of cardiovascular disease (31).

In conclusion, there was an increased risk of psychiatric disorders and cardiovascular disease during the diagnostic workup of suspected prostate cancer whether or not the workup led to a final cancer diagnosis.

## Funding

This study was supported by the Swedish Cancer Society (No. CAN 2017/322), the Swedish Research Council for Health, Working Life and Welfare (No. 2017–00531), and the Karolinska Institutet (Senior Researcher Award and Strategic Research Area in Epidemiology to Dr Fang Fang).

### Notes

**Role of the funder:** The funders had no role in the design of the study; the collection, analysis, and interpretation of the data; the writing of the manuscript; and the decision to submit the manuscript for publication

**Disclosures:** All authors have no conflict of interest to declare.

**Author contributions:** Conception and design: Qing Shen, Yuanjun Ma, Ove Andrén, Fang; Financial support: Fang; Administrative support: Fang; Provision of study materials or patients: Anna Jöud, Maria E. C. Schelin; Collection and assembly of data: Anna Jöud, Maria E. C. Schelin; Data analysis and interpretation: all authors; Manuscript writing: all authors; Final approval of manuscript: all authors; Accountable for all aspects of the work: all authors.

### Data Availability

Data from the study are available upon reasonable request. Permission for data sharing can be provided from fang.fang@ki.se.

## Supplementary Material

pkaa108_Supplementary_Data
