# Supplementary material for: Psychiatric Disorders and Cardiovascular Diseases During the Diagnostic Workup of Suspected Prostate Cancer
Source: JNCI Cancer Spectr. 2020 Nov 7;5(1):pkaa108. doi: 10.1093/jncics/pkaa108 (PMC7853179; doi:10.1093/jncics/pkaa108)

Psychiatric disorders and cardiovascular diseases during the diagnostic workup of suspected prostate cancer

Supplementary Materials

Supplementary Table 1: ICD codes for psychiatric disorders, and cardiovascular disease.

Psychiatric disorders	F10-F99
- Stress reaction or adjustment disorder	F43
- Depression	F32-F33
- Anxiety	F40-F41
- Substance abuse	F10-F19
Cardiovascular disease	I00-I99
- Myocardial infarction	I21, I22, I23, I24
- Other diseases of the heart	I10, I11, I12, I13, I71, I72
- Embolism or thrombosis	I26, I74, I81, I82
- Stroke	I60-I64

Supplementary Table 2: ICD codes used to identify possible prostate-related healthcare visits.

ICD-10 codes	Content
C61	Malignant neoplasm of prostate
D291	Benign neoplasm of male genital organs: prostate
D075	Carcinoma in situ of other and unspecified genital organs: prostate
D400	Neoplasm of uncertain or unknown behaviour of male genital organs: prostate
D409	Neoplasm of uncertain or unknown behaviour of male genital organs: male genital organ, unspecified
D41	Neoplasm of uncertain or unknown behaviour of urinary organs
N30	Cystitis
N39	Other disorders of urinary system
N40	Hyperplasia of prostate
N41	Inflammatory diseases of prostate
N42	Other disorders of prostate
N510	Disorders of prostate in diseases classified elsewhere
M544	Lumbago with sciatica
M545	Low back pain
M549	Dorsalgia, unspecified
M791	Other soft tissue disorders, not elsewhere classified: myalgia
K409	Unilateral or unspecified inguinal hernia, without obstruction or gangrene
R10	Abdominal and pelvic pain
R30	Pain associated with micturition
R31	Unspecified haematuria
R33	Retention of urine
R35	Polyuria
R39	Other symptoms and signs involving the urinary system
R52	Pain, not elsewhere classified
R53	Malaise and fatigue

Supplementary Table 3. Incidence rates (IRs, per 1000 person-months) and incidence rate ratios (IRRs) of psychiatric disorders and cardiovascular disease during the period before diagnosis of men who had a prostate diagnostic workup, according to age, calendar period, cohabitating status, and preexisting psychiatric disorder or cardiovascular disease, a population-based cohort study during 2005-2014 in Skåne, Sweden.

	Reference group [a]			Biopsy group			Prostate cancer patients		
	N	Crude IR	IRR (95% CI)†	N	Crude IR	IRR (95% CI) [b]	N	Crude IR	IRR (95% CI) [b]
PSYCHIATRIC DISORDERS									
Age in years									
18-39	312,020	11.89	1.00	55	36.18	2.05 (1.57-2.70)	0	0.00	-
40-49	126,747	13.90	1.00	118	51.25	2.37 (1.93-2.91)	12	46.40	3.08 (1.51-6.26)
50-59	97,787	11.77	1.00	258	39.46	2.22 (1.94-2.54)	57	20.02	1.54 (1.18-2.02)
60-69	44,957	8.09	1.00	340	26.07	2.42 (2.15-2.73)	150	19.37	2.12 (1.78-2.53)
70-79	19,747	7.53	1.00	194	20.06	2.28 (1.95-2.67)	99	18.48	2.35 (1.89-2.93)
80 and above	6,606	6.69	1.00	86	20.35	2.60 (2.04-3.31)	25	12.67	1.90 (1.28-2.81)
Calendar period									
2005-2009	311,773	6.55	1.00	349	19.29	2.64 (2.36-2.96)	133	13.80	2.26 (1.90-2.69)
2010-2014	296,091	56.82	1.00	702	36.56	1.61 (1.49-1.75)	210	24.55	1.34 (1.15-1.56)
Cohabitating status									

Cohabitating	172,268	7.90	1.00	517	21.98	2.45 (2.23-2.68)	178	14.37	1.97 (1.69-2.31)
Non-cohabitating	435,570	14.04	1.00	534	38.76	2.05 (1.87-2.25)	165	28.46	1.82 (1.54-2.15)
Preexisting psychiatric disorder									
Yes	507,176	66.79	1.00	847	129.01	2.11 (1.96-2.27)	302	122.94	2.06 (1.82-2.32)
No	100,688	2.23	1.00	204	6.64	2.87 (2.50-3.29)	41	2.61	1.13 (0.83-1.53)
CARDIOVASCULAR DISEASE									
Age in years									
18-39	72,377	2.76	1.00	34	22.37	4.16 (2.92-5.93)	0	0.00	-
40-49	119,878	13.15	1.00	92	39.94	2.67 (2.15-3.30)	10	38.67	3.27 (1.75-6.13)
50-59	243,143	29.27	1.00	516	78.90	2.76 (2.51-3.04)	162	56.93	2.34 (2.00-2.75)
60-69	282,236	50.78	1.00	1,634	125.25	2.61 (2.48-2.74)	703	90.78	2.14 (1.98-2.30)
70-79	207,144	79.00	1.00	1,798	185.90	2.44 (2.32-2.56)	739	138.11	2.04 (1.89-2.20)
80 and above	94,738	95.88	1.00	1,108	262.24	2.50 (2.35-2.66)	439	223.41	2.41 (2.19-2.66)
Calendar period									
2005-2009	556,239	12.07	1.00	2,010	111.17	2.57 (2.46-2.69)	913	94.87	2.42 (2.27-2.59)
2010-2014	463,277	68.59	1.00	3,172	164.98	2.40 (2.31-2.49)	1,140	133.35	1.98 (1.86-2.10)
Cohabitating status									
Cohabitating	623,915	28.61	1.00	3,253	138.29	2.53 (2.44-2.62)	1,329	107.33	2.16 (2.05-2.29)
Non-cohabitating	395,586	12.75	1.00	1,929	139.94	2.60 (2.48-2.72)	724	125.03	2.31 (2.14-2.49)

Preexisting cardiovascular disease									
Yes	888,034	84.93	1.00	4,921	255.49	2.61 (2.53-2.69)	1,890	222.46	2.23 (2.13-2.34)
No	131,482	3.10	1.00	261	14.46	1.91 (1.69-2.16)	163	16.84	1.87 (1.60-2.18)

[a] Reference group included person-time accumulated from men who did not have any prostate diagnostic workup during the follow-up and the person-time accumulated before the start of workup from men with a prostate diagnostic workup during the follow-up.

[b] CI, confidence interval. All the analyses were adjusted for age, cohabitating status, registered parish, and preexisting psychiatric disorders or cardiovascular disease.

Supplementary Table 4 Incidence rates (IRs, per 1000 person-months) and incidence rate ratios (IRRs) of psychiatric disorders and cardiovascular disease during the period before diagnosis of men who underwent a prostate diagnostic workup, with additional adjustment for frequency of healthcare visit, a population-based cohort study during 2005-2014 in Skåne, Sweden.

	No. of events	Crude IR	IRR (95% CI)[a]	IRR (95% CI)[b]
PSYCHIATRIC DISORDERS				
Reference group	607,864	11.50	1.00	1.00
Biopsy group	1,051	28.18	2.22 (2.08-2.37)	1.70 (1.59-1.83)
Prostate cancer patients	343	18.86	1.87 (1.67-2.10)	1.32 (1.16-1.50)
CARDIOVASCULAR DISEASES				
Reference group	1,019,516	19.30	1.00	1.00
Biopsy group	5,182	138.90	2.56 (2.49-2.63)	2.04 (1.97-2.11)
Prostate cancer patients	2,053	112.97	2.22 (2.12-2.32)	1.67 (1.59-1.75)

[a] Estimates were adjusted for age, cohabitating status, registered parish, and preexisting psychiatric disorders or cardiovascular disease.
[b] Estimates were adjusted for [a], with additional adjustment for frequency of healthcare visits per time period.

Supplementary Table 5. Incidence rates (IRs, per 1000 person-months) and incidence rate ratios (IRRs) of psychiatric disorders and cardiovascular disease during the period before biopsy for men who had repeated biopsies during 2005-2014 in Skåne, Sweden, a within-individual comparison.

	No. of events	Crude IR	IRR (95% CI) [b]
PSYCHIATRIC DISORDERS			
Reference period [a]	11,959	9.79	1.00
Period before 2nd biopsy [c]	310	20.78	1.60 (1.36-1.87)
Period before 3rd biopsy [c]	113	16.97	1.37 (1.08-1.73)
Period before 4th biopsy [c]	54	18.03	1.53 (1.06-2.23)
CARDIOVASCULAR DISEASE			
Reference period [a]	49,337	40.40	1.00
Period before 2nd biopsy [c]	1,387	92.98	1.44 (1.35-1.54)
Period before 3rd biopsy [c]	561	84.26	1.26 (1.14-1.40)
Period before 4th biopsy [c]	239	79.78	1.23 (1.06-1.44)

[a] Reference group included person-time accumulated before the start of the first diagnostic workup from men with repeated biopsies during the follow-up.

[b] CI, confidence interval. Estimates were additionally adjusted for preexisting psychiatric disorder or cardiovascular disease.

[c] The start of period before the 2nd, 3rd and 4th biopsy was defined as the first healthcare visit within three months before the 2nd, 3rd and 4th biopsy, or the 90th day before the corresponding biopsy, the same as in the main analysis (36.1% of men with a 2nd biopsy, 43.8% with a 3rd biopsy and 48.3% with a 4th biopsy were assigned 90th day as start of workup).

Supplementary Figure 1. Weekly frequency of healthcare visits before and after the date of biopsy for prostate cancer patients (A) and men in biopsy group (B), a population-based cohort study during 2005-2014 in Skåne, Sweden.*

*The weekly frequency of all healthcare visits during the 16 weeks before and 8 weeks after diagnosis for all men who had a diagnostic workup of prostate cancer during follow-up.

A. Prostate cancer patients

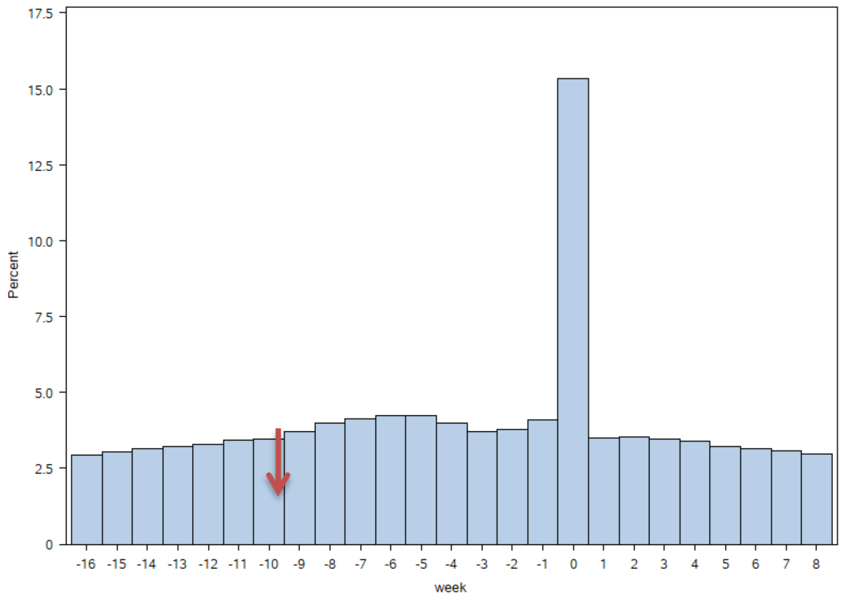

B. Men in biopsy group

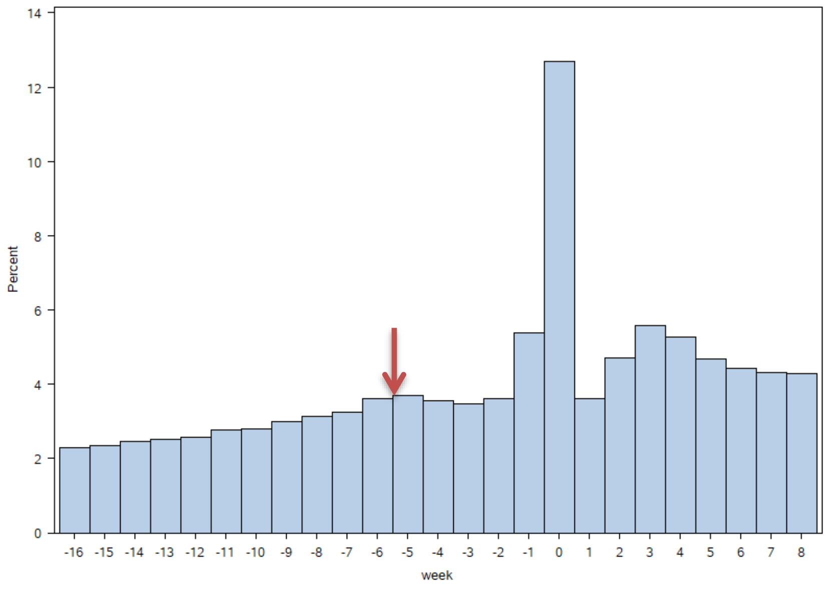

Supplement: pkaa108_Supplementary_Data [file pkaa108_supplementary_data.pdf]
